# Prognostic value of ambulation ability with albumin and C-reactive protein to predict 28-day mortality in elderly sepsis patients: a retrospective multicentre registry-based study

**DOI:** 10.1186/s12877-022-03339-2

**Published:** 2022-08-12

**Authors:** Kyungman Cha, Seung Pill Choi, Soo Hyun Kim, Sang Hoon Oh

**Affiliations:** 1 Department of Emergency Medicine, Suwon St. Vincent Hospital, College of Medicine, The Catholic University of Korea, Seoul, Republic of Korea; 2 Department of Emergency Medicine, Eunpyeong St. Mary’s Hospital, College of Medicine, The Catholic University of Korea, 1021 Tongil Blvd., Eunpyeong, Seoul, Republic of Korea 03312; 3 Department of Emergency Medicine, Seoul St. Mary’s Hospital, College of Medicine, The Catholic University of Korea, Seoul, Republic of Korea

**Keywords:** Aged, Sepsis, Walking, Albumins, C-Reactive Protein

## Abstract

**Background:**

Sepsis is a series of organ failures caused by dysregulated responses to infection. Risk factors for sepsis are multiple comorbidities, a poor nutrition status, and limited mobility. The primary purpose of the study was to determine whether ambulation ability with albumin and C-reactive protein are predictive of 28-day mortality of elderly patients with sepsis.

**Methods:**

This was a retrospective observational study using a multicentre-based registry of elderly patients between November 2016 and February 2017. The inclusion criteria were a patient ≥65 years and a diagnosis of sepsis and exclusion criteria were a patient with covariates of ambulation ability such as central nervous system diseases, or malignancy. The area under the receiver operating characteristic curve of prediction models were calculated and compared. The survival rates according to the ambulation ability were estimated and compared by the log-rank test.

**Results:**

2291 patients ≥65 years visited with infectious diseases. 496 subjects with central nervous system diseases, 710 subjects with malignancy and 817 subjects with a Sequential Organ Failure Assessment score ≤ 1 were excluded. Ultimately, 278 subjects were included in the primary analysis. 133 (47.8%) subjects were male and the median age was 78 years. 228 (82%) subjects could ambulate independently before morbidity and 28 (10.1%) subjects expired in 28 days. In the inability to ambulate and C-reactive protein to albumin ratio model, the area under the curve predicting 28-day mortality was 0.761 with no significant difference from the Sequential Organ Failure Assessment score (0.859, *p* = 0.097) and the estimated survival rate on 28th day according to the ability to ambulate showed a significant difference (hazard ratio = 1.212, *p* < 0.001).

**Conclusion:**

The premorbid ambulation ability with albumin and C-reactive protein can be combined to predict 28-day mortality in elderly patients with sepsis.

**Supplementary Information:**

The online version contains supplementary material available at 10.1186/s12877-022-03339-2.

## Background

Sepsis is a series of fatal organ failures caused by dysregulated responses to infection, and more than 30 million people are affected by this disease each year worldwide. Approximately 5 million people are estimated to die from this illness, and the incidence is increasing. Known risk factors for sepsis are multiple comorbidities, poor nutrition status, compromised immune system and functional incapacity, such as limited mobility; these factors can cause adverse outcomes and are more common in older people, especially those who are frail [1–4].

Ageing is usually accompanied by gradual changes in physical and cognitive function and changes in the state of existing underlying diseases [5]. Once an individual reaches a certain stage, they are more likely to worsen than to recover from external stress events. This stage is defined as frailty, and numerous efforts have been made for many years to clinically describe and quantify vulnerability [6–13]. In addition, frailty is highly related to the prognosis of cardiovascular disease and malignancy, and its usefulness as a selection criterion for treatment modalities and a tool for risk stratification and clinical outcome prediction has been supported by many studies [14–16]. Quantitative criteria that have been used to evaluate frailty include the number of underlying diseases, nutritional status, body weight change, grip strength, daily time spent standing and being active, gait speed, ability to climb a ramp or stairs, cognitive ability, and presence of neuropsychiatric diseases [6, 8, 10, 11, 17, 18].

C-reactive protein, which is produced in the liver and is a key inflammatory mediator against bacterial infection and sepsis, has been widely used as an indicator of response to infection in clinical fields since it was found by Tillet in 1930; additionally, a low serum albumin level has been known to be significantly associated with susceptibility to bacterial infections and declining function in elderly people [19].

The primary purpose of this study was to determine whether ambulation ability with albumin as a measure of nutritional status and C-reactive protein as an indicator of response to sepsis are predictive of 28-day mortality in elderly patients diagnosed with sepsis.

## Methods

### Study design and patients

This was a retrospective observational study using a multicentre-based registry of elderly patients and the standardized digital medical records of three emergency medical centres at urban academic hospitals, visited by more than 60,000 patients annually. Data from patients who visited the emergency department (ED) from 1 November 2016 to 28 February 2017, were reviewed.

The inclusion criteria were patients aged older than 65 years with a primary diagnosis of infectious diseases or sepsis on the discharge record. Sepsis was defined as an increase of 2 points or more in the Sequential Organ Failure Assessment (SOFA) score at the time of admission.

We excluded patients with covariates of ambulation ability such as history of stroke, Parkinson’s disease, cognitive disorders (including Alzheimer’s disease), metabolic encephalopathy, epilepsy, musculoskeletal disease, or malignancy.

### Data collection

Demographic data were collected, including patient age and sex; vital signs; mental status according to the Glasgow Coma Scale (GCS) score; past medical history of diabetes, hypertension, coronary artery disease, heart failure, chronic liver disease, chronic kidney disease, chronic lung disease, peptic ulcer disease and arthritis; ambulation ability prior to morbidity; type of residence before ED presentation including nursing facility; laboratory test results; endotracheal intubation and use of mechanical ventilation; central venous catheterization and vasopressor therapy; length of hospital stay; intensive care unit (ICU) admission; in-hospital mortality; 28-day mortality; and transfer to other medical facilities.

As a well-known mortality prediction score, the Charlson comorbidity index (CCI), a predictor of mortality in sepsis, the quick SOFA (qSOFA) score and the SOFA score were assessed from ED medical records and laboratory test results. The patient’s ambulation ability was defined as the ability to walk independently without help from others, with or without the use of a cane or a walker. In the evaluation of ambulation ability, gait speed and lower limb amputation were not considered. According to emergency medical records, admission notes, nursing records, consultation notes from the neurology or neurosurgery departments and digitally scanned transfer records, patients who were bedridden or required a wheelchair for mobility were considered unable to walk.

### Statistical analysis

Continuous variables were calculated as the means ± standard deviations if they had a normal distribution or as medians if not, and categorical variables were calculated as percentages. χ^2^ and Mann-Whitney U tests were used to compare the proportion and distribution of variables between the survivor and nonsurvivor groups.

To evaluate the predictive value of the variables, those with significance on univariate analysis were included in a multivariate logistic regression, and the odds ratios (ORs) and 95% confidence intervals (CIs) were estimated. A linear regression was performed to determine the effect of the laboratory variables on 28-day mortality, and variables with a variance inflation factor (VIF) greater than 4.0 were excluded from further regression analysis.

We also created combined models using several logistic regressions that included the variables related to primary outcomes, and the area under the receiver operating characteristic (ROC) curve (AUC), sensitivity and specificity of each prediction model were calculated. To compare the accuracy of the outcome prediction, pairwise AUC comparisons were performed between two prediction models using the nonparametric approach developed by DeLong et al. The 28-day survival rates of the prediction models were estimated by Kaplan-Meier analysis, and the difference between the models was determined using the log-rank test. All statistical analyses were performed using SPSS version 22 (SPSS, Inc., Chicago, IL, USA) and MedCalc 12.0 (MedCalc Software Inc., Mariakerke, Belgium). A *p*-value <0.05 was considered statistically significant.

## Results

### Study population

**Fig. 1:**
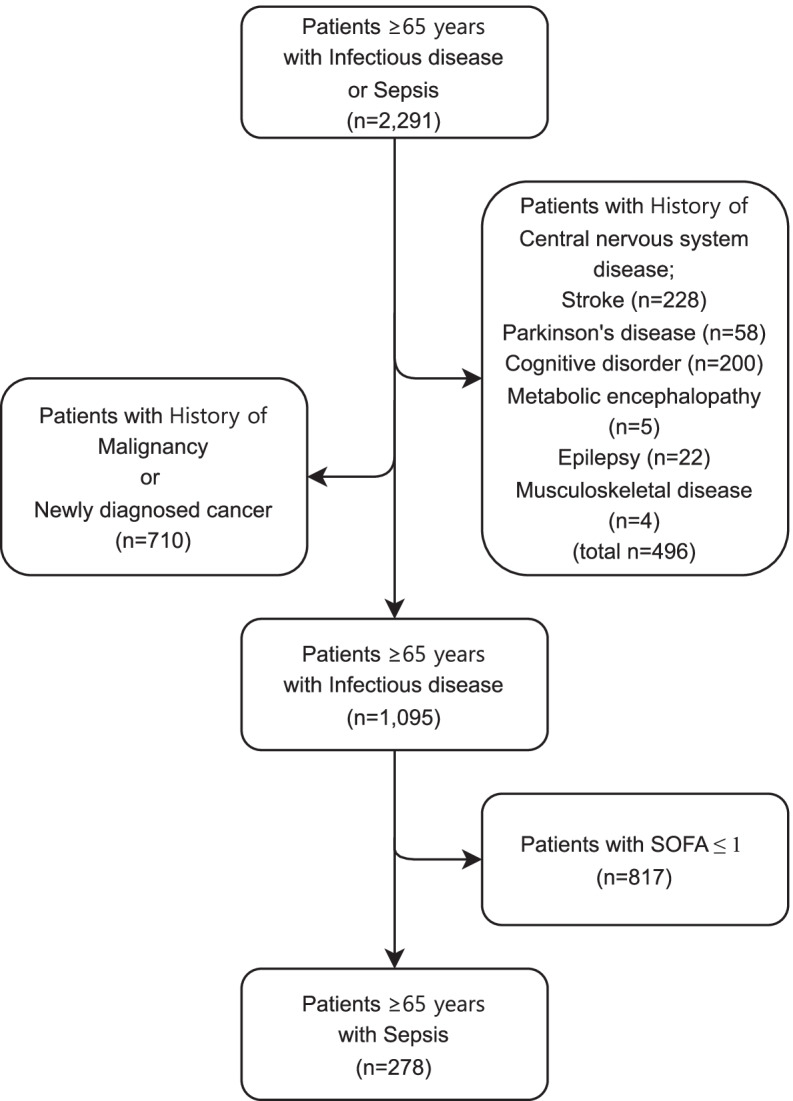
The process of determining the research subjects; patients ≥65 years with sepsis presenting the emergency department

During the study period, 2291 patients who were older than age 65 and had a primary diagnosis of infectious diseases or sepsis on their discharge records visited the ED. A total of 228 subjects with a history of stroke were excluded, along with 58 subjects with Parkinson’s disease; 200 subjects with cognitive disorders, including Alzheimer’s disease; 5 subjects with metabolic encephalopathy; 22 subjects with epilepsy; 4 subjects with musculoskeletal disease; 710 subjects who had a history of or newly diagnosed malignancy; and 817 subjects with a SOFA score of 1 or less. Ultimately, 278 subjects were included in the primary analysis (Fig. 1).

A total of 133 (47.8%) subjects were male, 145 (52.2%) were female, and the median age was 78 years (interquartile range [IQR], 71–83). A total of 188 (67.6%) subjects arrived from their home, 70 (25.2%) were transferred from another medical institution, and 20 (7.2%) were transferred from a nursing hospital or nursing home. A total of 228 (82%) subjects could ambulate independently before ED presentation.

A total of 124 (44.6%) subjects had infections of respiratory origin, including influenza, community-acquired pneumonia, or aspiration pneumonia; 59 (21.2%) had urinary tract infections; 19 (6.8%) had gastrointestinal infections; 41 (14.7%) had hepatobiliary infections; 13 (4.6%) had soft tissue and musculoskeletal infections; and 73 (26.3%) had a positive result from blood culture tests. A total of 235 (84.5%) subjects were admitted to the hospital, 79 (28.4%) subjects received treatment in the ICU, and 40 (14.4%) subjects required mechanical ventilation. Thirty-five (12.6%) subjects died in the hospital during treatment, and 28 (10.1%) subjects expired within 28 days (Table 1).

**Table 1:** Demographic and clinical characteristics of the subjects

		Nonsurvivors (*n* = 28)	Survivors (*n* = 250)
Sex, male	133 (47.8)	11 (39.3)	122 (48.8)
Age (years)	78 (71–83)	79.5 (70.3–83.3)	78 (75–83)
Departure place for ED^a)^ visit			
Home	188 (67.6)	18 (64.3)	170 (68)
Other medical centres	70 (25.2)	8 (28.6)	62 (24.8)
Nursing hospital or facility	20 (7.2)	2 (7.1)	18 (7.2)
Inability to ambulate	50 (18)	5 (35.7)	45 (16.0)
Medical history			
Diabetes mellitus	105 (37.8)	13 (46.4)	92 (36.8)
Hypertension	177 (63.7)	17 (60.7)	160 (64.0)
Coronary artery disease	40 (14.4)	4 (14.3)	36 (14.4)
Congestive heart failure	24 (8.6)	4 (14.3)	20 (8.0)
Chronic liver disease	10 (3.6)	0 (0)	10 (4)
Chronic renal disease	37 (13.3)	4 (14.3)	33 (13.2)
Chronic lung disease	84 (30.2)	11 (39.3)	73 (29.2)
Peptic ulcer disease	9 (3.2)	2 (7.1)	7 (2.8)
Arthritis	76 (27.3)	11 (39.3)	65 (26.0)
Charlson comorbidity index	4 (4–5)	4 (4–6)	4 (4–5)
Diagnosis of infectious diseases			
Respiratory	124 (44.6)	19 (67.9)	105 (42.0%)
Urinary tract	59 (21.2)	3 (10.7)	56 (22.4)
Gastrointestinal	19 (6.8)	3 (10.7)	16 (6.4)
Hepatobiliary	41 (14.7) 9 (18.1)	0 (0.0)	41 (16.4)
Soft tissue and musculoskeletal	13 (4.6)	3 (10.7)	10 (4.0)
Others^b)^	22 (7.9)	0 (0.0)	22 (8.8)
Positive blood culture	73 (26.3)	10 (35.7)	63 (25.2)
Admission in Intensive Care Units (ICU)	79 (28.4)	20 (71.4)	59 (23.6)
Managements			
Intubation	42 (15.1)	18 (64.3)	24 (9.6)
Ventilator care	40 (14.4)	17 (60.7)	23 (9.2)
Central venous catheter	53 (19.1)	17 (60.7)	36 (14.4)
Vasopressor infusion	61 (21.9)	20 (71.4)	41 (16.4)
Septic shock^c)^	38 (13.7)	14 (50)	24 (9.6)
Length of stay in ICU^d)^ (days)	4 (2–8)	3.5 (2.0–7.5)	5 (3–9)
Length of stay in hospital (days)	10 (5–16)	5 (2.8–13.3)	10 (6–16)

Variables are expressed as n (%) or median (interquartile range)

^a)^*ED* Emergency department

^b)^Others. Rickettsia infection, central nervous system infection and unknown origin

^c)^Use of vasopressors to maintain mean arterial pressure of 65 mmHg and a lactate level > 2 mmol/L despite volume resuscitation

^d)^*ICU* Intensive care unit

### Correlation of inflammatory biomarkers and mortality prediction scores with 28-day mortality

The distribution of the laboratory values, including inflammatory biomarkers and mortality prediction scores such as the white blood cell (WBC) count, lactate, C-reactive protein (CRP), and albumin levels as indicators of nutritional status, CRP-to-albumin ratio (CAR), and qSOFA and SOFA scores were compared between the survivor and nonsurvivor groups. The CRP level, albumin level, CAR, and qSOFA and SOFA scores were significantly related to 28-day mortality (*p* < 0.001, *p* = 0.001, *p* < 0.001, *p* = 0.026 and *p* < 0.001, respectively) (Table 2).

**Table 2:** The comparison of laboratory values and mortality prediction scores between nonsurvivors and survivors

	Nonsurvivors (*n* = 28)	Survivors (*n* = 250)	*p*-value
WBC^a)^ count (×10^9^/L)	12.9 (5.2–18.3)	11.9 (8.0–16.6)	0.900
Lactate^b)^ (mmol/L)	3.6 (1.9–6.7)	1.6 (1.1–2.7)	<0.000
C-reactive protein (mg/dL)	18.3 (9.5–26.7)	8.4 (3.9–17.7)	<0.001
Albumin (g/dL)	3.0 (2.8–3.4)	3.5 (3.0–3.8)	0.001
CRP^c)^ to albumin ratio	6.1 (2.8–9.5)	2.4 (1.0–5.3)	<0.001
qSOFA^d)^ score			0.026
0–1	18 (64.3)	205 (82.0)	
≥ 2	10 (35.7)	45 (18.0)	
SOFA^e)^ score	7 (5.3–8.8)	3 (2–4)	<0.001

Variables are expressed as n (%) or median (interquartile range)

^a)^*WBC* White blood cell

^b)^*n* = 232

^c)^*CRP* C-reactive protein

^d)^*qSOFA* Quick Sequential Organ Failure Assessment

^e)^*SOFA* Sequential Organ Failure Assessment

In the univariate analysis, an inability to ambulate, respiratory infection, CRP level, albumin level and CAR were significantly correlated with the 28-day mortality rate (OR = 2.917, 95% CI 1.254–6.781, *p* = 0.013; 2.915, 1.269–6.699, *p* = 0.012; 1.078, 1.036–1.123, *p* < 0.001; 0.353, 0.187–0.668, *p* = 0.001; 1.281, 1.146–1.431, and *p* < 0.001). In the linear regression analysis, the CRP level and CAR showed collinearity for the 28-day mortality rate (VIF = 17.549 and 21.557); then, the CAR was excluded from the subsequent regression. In a multivariate logistic regression, respiratory infection and CRP level were found to be predictive of 28-day mortality (2.594, 1.060–6.348, *p* = 0.037; 1.076, 1.032–1.123, and *p* = 0.001) (Table 3).

**Table 3:** Logistic regression analysis for 28-day mortality

	Univariate		Multivariate	
	OR^b)^ (95% CI^c)^)	*p*-value	OR^b)^ (95% CI^c)^)	*p*-value
Inability to ambulate	2.917 (1.254–6.781)	0.013	1.892 (0.650–5.504)	0.224
Respiratory infection	2.915 (1.269–6.699)	0.012	2.594 (1.060–6.348)	0.037
Hepatobiliary infection	0.000	0.998	N/A^d)^	N/A^d)^
C-reactive protein	1.078 (1.036–1.123)	<0.001	1.076 (1.032–1.123)	0.001
Albumin	0.353 (0.187–0.668)	0.001	0.559 (0.251–1.244)	0.154
CRP^a)^ to albumin ratio	1.281 (1.146–1.431)	<0.001	N/A^d)^	N/A^d)^

^a)^*CRP* C-reactive protein

^b)^*OR* Odds ratio

^c)^*CI* Confidence interval

^d)^*N/A* Not applicable

### Pairwise AUC comparisons between models to measure index accuracy in predicting 28-day mortality

**Fig. 2:**
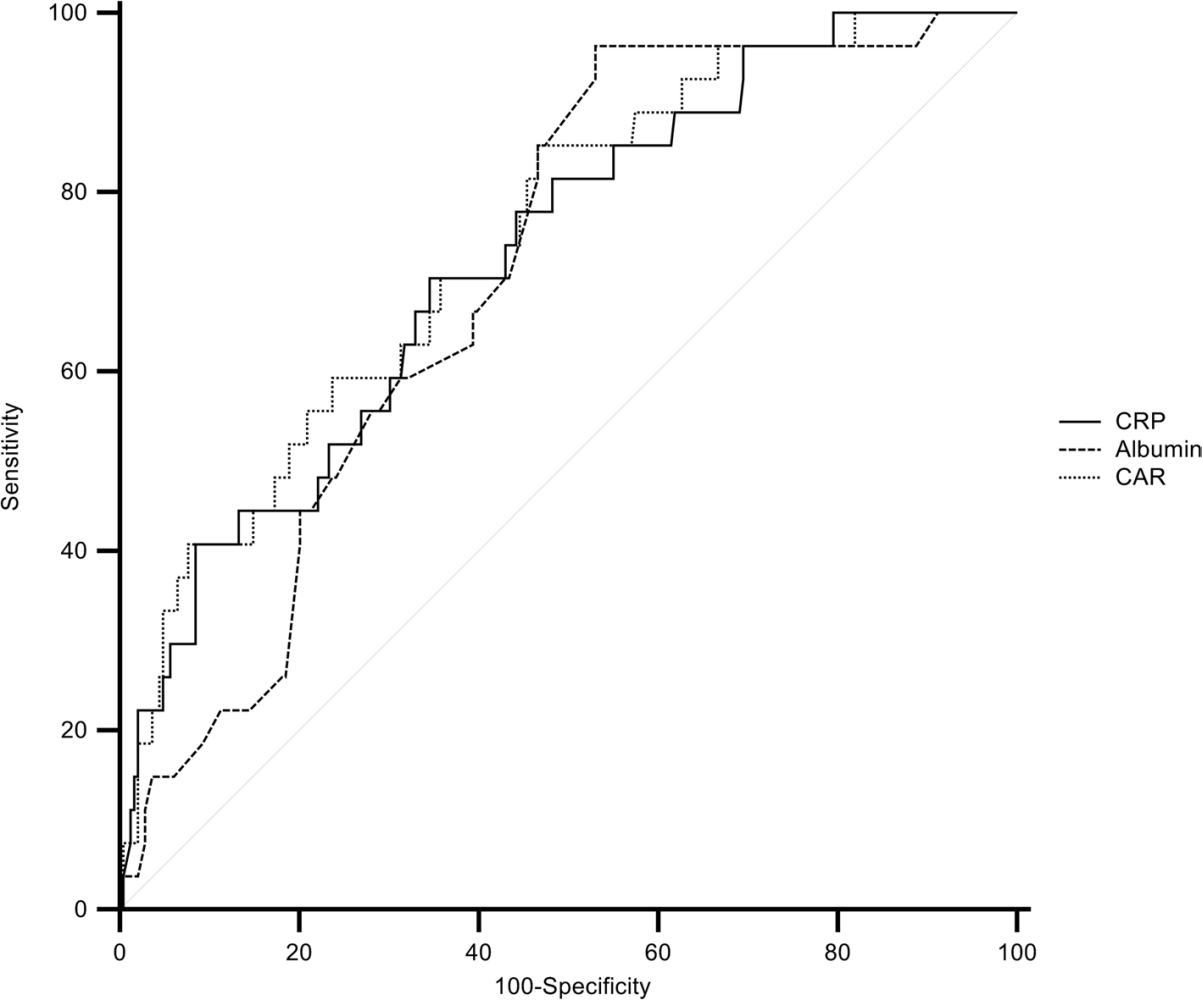
Comparison of receiver operating characteristic curve of CRP, albumin and CAR for the prediction of 28-day mortality. CRP: C-reactive protein; CAR: C-reactive protein to albumin ratio

**Fig. 3:**
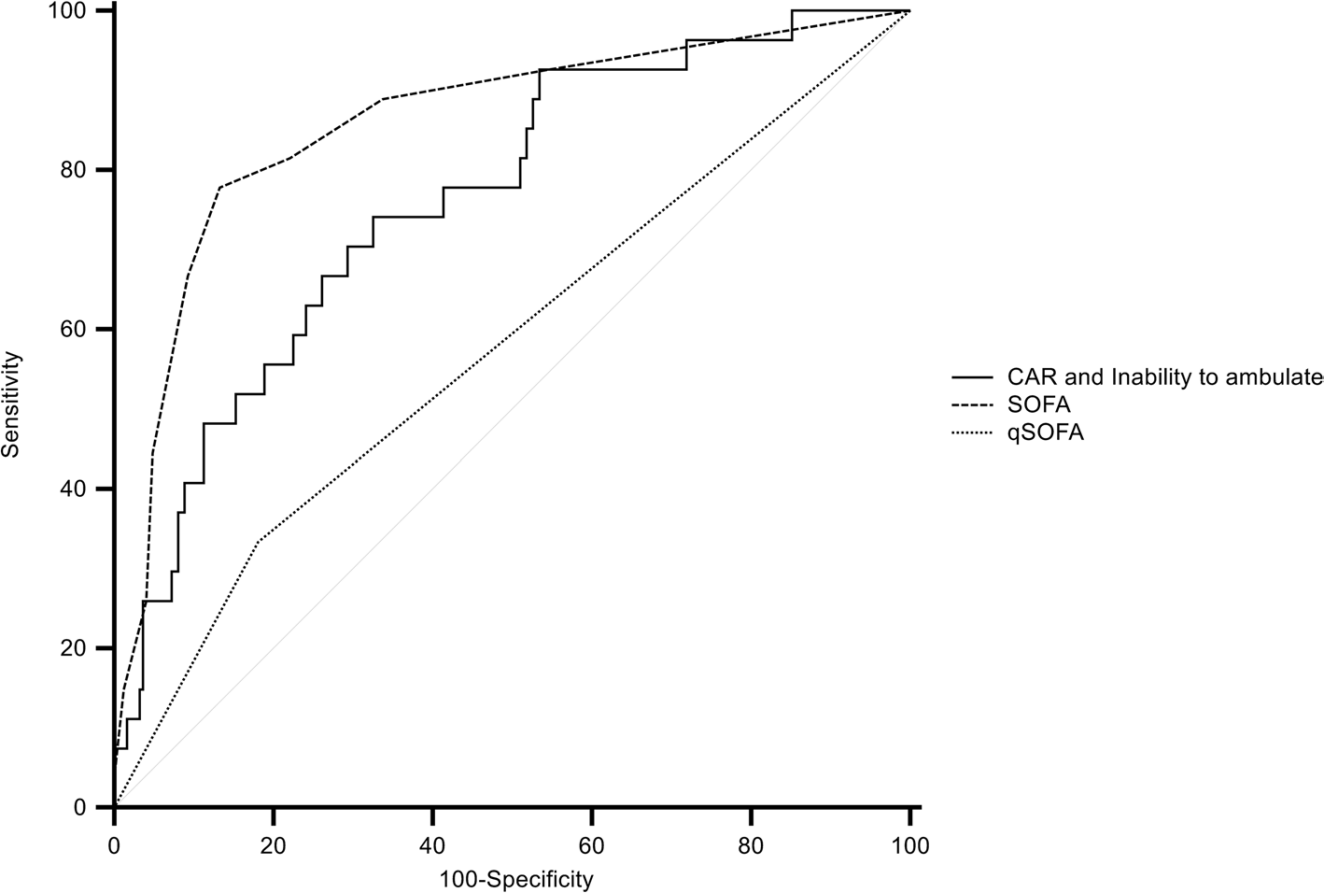
Comparison of receiver operating characteristic curve of CAR and inability to ambulate model, SOFA and qSOFA scores for the prediction of 28-day mortality. CAR: C-reactive protein to albumin ratio; SOFA: Sequential Organ Failure Assessment; qSOFA: quick Sequential Organ Failure Assessment

There was no statistically significant difference in the ability of the CRP level, albumin level, or CAR to predict 28-day mortality, according to the AUC values (Table 4, Fig. 2). The AUC values of predictive models made with an inability to ambulate and the above variables were compared by multivariate ROC analysis (Supplementary Tables 1, 2), and in the model of CAR and an inability to ambulate, which was independent of the SOFA score, the AUC predicting 28-day mortality was 0.761 (95% CI 0.706–0.810), indicating no statistically significant difference from the SOFA score (*p* = 0.097) (Table 5, Fig. 3).

**Table 4:** Comparison of area under the curve between variables related to 28-day mortality

	AUC^b)^ (95% CI^c)^)	*P*-value between variables		
		CRP^a)^ to albumin ratio	Albumin	C-reactive protein
C-reactive protein	0.730 (0.674–0.782)	0.182	0.686	
Albumin	0.709 (0.651–0.762)	0.391		
CRP^a)^ to albumin ratio	0.747 (0.691–0.797)			

^a)^*CRP* C-reactive protein

^b)^*AUC* Area under the curve

^c)^*CI* Confidence interval

**Table 5:** Comparison of area under the curve between prediction model and mortality prediction scores for 28-day mortality

	AUC^d)^ (95% CI^e)^)	*P*-value between predictors		
		SOFA^c)^	qSOFA^b)^	CRP^a)^ to albumin ratio and inability to ambulate
CRP^a)^ to albumin ratio and inability to ambulate	0.761 (0.706–0.810)	0.097	0.003	
qSOFA^b)^	0.589 (0.528–0.647)	<0.001		
SOFA^c)^	0.859 (0.813–0.898)			

^a)^*CRP* C-reactive protein

^b)^*qSOFA* quick Sequential Organ Failure Assessment

^c)^*SOFA* Sequential Organ Failure Assessment

^d)^*AUC* Area under the curve

^e)^*CI* Confidence interval

**Fig. 4:**
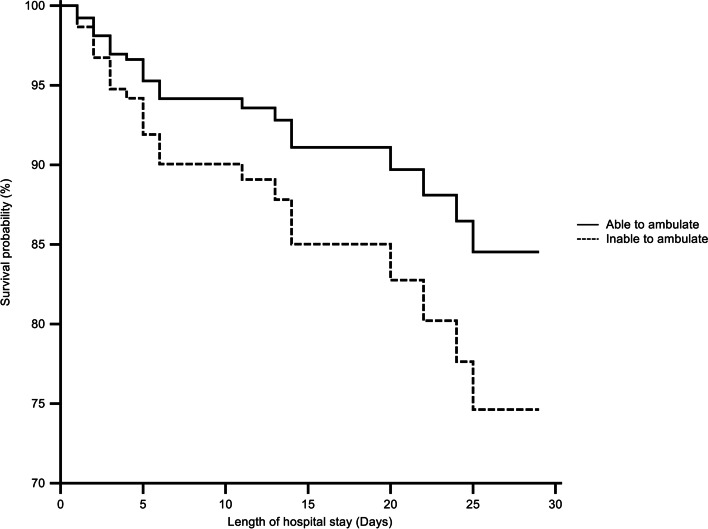
Kaplan-Meier survival curve of the model including CAR according to the ability to ambulate

In the univariate Cox regression analysis, an inability to ambulate, respiratory infection, CRP level, albumin level and CAR were found to have statistically significant effects on the 28-day survival rate (*p* = 0.031, *p* = 0.043, *p* < 0.001, *p* = 0.013 and *p* < 0.001). Through multivariate Cox regression, in the model including the CAR, the estimated survival rate on the 28th day according to the ability to ambulate showed a statistically significant difference (hazard ratio = 1.212, 95% CI 1.114–1.319, *p* < 0.001) (Table 6, Fig. 4).

**Table 6:** Cox regression analysis for estimation of the survival rate on the 28th day according to the ability to ambulate

	Univariate		Multivariate	
	HR^b)^ (95% CI^c)^)	*p*-value	HR^b)^ (95% CI^c)^)	*p*-value
Inability to ambulate	2.352 (1.083–5.108)	0.031	N/A	N/A
Respiratory infection	2.274 (1.027–5.033)	0.043	2.077 (0.935–4.613)	0.022
C-reactive protein	1.057 (1.029–1.086)	<0.001	1.061 (1.030–1.093)	<0.001
Albumin	0.468 (0.257–0.851)	0.013	0.542 (0.281–1.042)	0.023
CRP^a)^ to albumin ratio	1.219 (1.119–1.328)	<0.001	1.212 (1.114–1.319)	<0.001

^a)^*CRP* C-reactive protein

^b)^*HR* Hazard ratio

^c)^*CI* Confidence interval

## Discussion

In this study, in elderly patients with sepsis, an inability to ambulate before hospitalization had a significant positive correlation with 28-day mortality, but it was not a predictive factor (χ^2^ = 4.905, *p* = 0.010; OR = 1.892, 95% CI 0.650–5.504, and *p* = 0.224). Hospital admission for sepsis itself is a known negative consequence of the disease [1], and in the study group, in which the majority (*n* = 235, 84.5%) of the patients were admitted for inpatient treatment, it is possible that the statistical effect of an inability to ambulate on mortality was reduced.

In previous studies, the mortality rate for severe sepsis was reported to be 20–30%, and in recent studies, the in-hospital mortality rate was less than 20% and has been decreasing [1, 2]. The present study found an in-hospital mortality rate of 12.6% (*n* = 35), similar to recent studies; therefore, the participating hospitals and emergency medical centres seem to be providing adequate management for sepsis patients.

Although the qSOFA score was significantly higher in the nonsurvivor group (*p* = 0.026), the AUC predicting 28-day mortality showed a low discriminative ability (0.589, 95% CI 0.528–0.647). The reasons are assumed to be that the patients included in the analysis were already classified as having a score ≥ 2 based on the SOFA system, which is a superior and definitive scoring system for predicting adverse outcomes in sepsis patients, and that the median value of the GCS score, which is one of the elements of the qSOFA, was 15 (IQR 15–15), and most patients (242, 87.1%) had a GCS score of 15 points, which seems to be due to the exclusion of patients with central neurological diseases with covariates of ambulation ability before the primary analysis.

In a previous study by Ayranci MK et al. of 784 elderly patients who visited the ED (median age 75, excluding patients with haematologic malignancy and traumatic injury), the CAR was correlated with in-hospital mortality (*n* = 78, *p* < 0.001) and the AUC of the CAR in predicting mortality was 0.723, which was similar to the value found in our study (0.747) [20]. In a study by Basile-Filho A et al. of 847 surgical patients admitted to the ICU (median age 62), the CAR was correlated with in-hospital mortality (*n* = 82, *p* < 0.001), and the AUC of the CAR in predicting mortality was 0.731. Additionally, similar to our study, the CAR was correlated with surgical sepsis-related morbidity during the postoperative period (*n* = 83, median age 62, *p* < 0.001), but the CAR alone could not predict mortality in surgical sepsis patients (*n* = 48, *p* = 0.709) [21].

Septic shock is defined as the state of sepsis that requires vasopressors to maintain a mean arterial pressure of 65 mmHg and a serum lactate level > 2 mmol/L despite adequate volume resuscitation [3]. In the study cohort, the ratio of septic shock patients was significantly higher in the nonsurvivor group (χ^2^ = 37.270, *p* < 0.001). In the group of septic shock patients, the proportion of patients who could not ambulate before morbidity was higher (13, 34.2% and 37, 15.4%, and *p* = 0.005) than that of the group of nonseptic shock patients, and this difference describes how an inability to ambulate detrimentally affects the prognosis of elderly patients with sepsis.

In the AUC comparison for predicting 28-day mortality, the model of CAR and an inability to ambulate did not show a statistically significant difference from the SOFA score (0.761 vs. 0.859, *p* = 0.097). Thus, evaluating the functional capacity combined with the nutritional status and levels of inflammatory biomarkers can have as much predictive power as evaluating the SOFA score in the study population.

Gait speed has been recognized as an important factor that defines frailty and as a criterion for determining frailty in several studies [6, 7, 9, 10, 13, 22–24]. The measurement of gait speed involves several variables, such as location, distance, position at the beginning of ambulation, surface condition, slope, path curvature, provision of verbal guidance, and management of falls during measurement. As an indicator of functional capacity before an illness, ambulation ability which is obtained from family members, relatives, or guardians in the ED, has a limited function as a tool to evaluate the degree of frailty, but it could have the advantage of being free from interference from the above factors and being easily applied in the ED [9, 13, 22–24].

Although ambulation ability was determined by using as many medical records as possible, the most important limitation of the study was that the subjects’ ability to walk a distance of 207 to 350 m required for a favourable prognosis of chronic cardiopulmonary diseases [25–27] or even a distance of 2.5–6 m (8–20 ft) required to exceed the domestic activity of daily living (ADL) and used in several studies [9, 10, 13, 23, 24] could not be confidently ascertained. This limitation might be the reason why the study showed that ambulation ability was related to mortality but was not predictive.

This was a retrospective observational study and was subject to selection bias and errors of documentation and data entry when the patient information was missing, or the medical records were incomplete. Our sample size was small, and there was a limit to the generalization of the results, a wide CI of statistical results or a nonnormal distribution, contrary to the assumption of the DeLong test in pairwise AUC comparisons. Thus, the conclusions of the study should be interpreted and confirmed through a prospective study with a large sample size.

## Conclusion

The premorbid ambulation ability with the albumin level as an indicator of nutritional status and the C-reactive protein level as an inflammatory biomarker can be combined to predict 28-day mortality in elderly patients diagnosed with sepsis and is not inferior to the SOFA score.

## Supplementary Information

**Additional file 1: Supplementary Table 1.** The area under the curve, sensitivity, and specificity of the best cut-off value of each predictive model related to 28-day mortality. **Supplementary Table 2.** Comparison of area under the curve between predictive models related to 28-day mortality.

## Data Availability

The datasets generated and/or analyzed during the current study are not publicly available due to privacy and ethical concerns, but are available from the corresponding author on reasonable request.

## Abbreviations

ADL: Activity of Daily Living
AUC: Area Under the Receiver Operating Characteristic Curve
CAR: C-Reactive Protein to Albumin Ratio
CCI: Charlson comorbidity index
CI: Confidence Interval
CRP: C-Reactive Protein
ED: Emergency Department
GCS: Glasgow Coma Scale
ICU: intensive care unit
IQR: Interquartile Range
OR: Odds Ratio
qSOFA: quick Sequential Organ Failure Assessment
ROC: Receiver Operating Characteristic
SOFA: Sequential Organ Failure Assessment
VIF: Variance Inflation Factor
WBC: White Blood Cell
