# Supplementary material for: Prognostic value of ambulation ability with albumin and C-reactive protein to predict 28-day mortality in elderly sepsis patients: a retrospective multicentre registry-based study
Source: BMC Geriatr. 2022 Aug 12;22:661. doi: 10.1186/s12877-022-03339-2 (PMC9373310; doi:10.1186/s12877-022-03339-2)
Supplement: Supplementary file 1 — Additional file 1: Supplementary Table 1. The area under the curve, sensitivity, and specificity of the best cut-off value of each predictive model related to 28-day mortality. Supplementary Table 2. Comparison of area under the curve between predictive models related to 28-day mortality. [file 12877_2022_3339_MOESM1_ESM.docx]

**Supplementary Tables**

Supplementary table 1. The area under the curve, sensitivity, and specificity of the best cut-off value of each predictive model related to 28-day mortality

|  | AUC^c)^ (95% CI^d)^) | Cut-off value | Sensitivity (95% CI^d)^) | Specificity (95% CI^d)^) |
| --- | --- | --- | --- | --- |
| CRP^a)^, albumin and inability to ambulate | 0.762 (0.707-0.811) | >0.05 | 96.3 (81.0-99.9) | 48.6 (42.2-55.0) |
| Albumin, CAR^b)^ and inability to ambulate | 0.763 (0.708-0.812) | >0.052 | 92.6 (75.7-99.1) | 49.0 (42.6-55.4) |
| CRP^a)^ and inability to ambulate | 0.750 (0.695-0.800) | >0.12 | 59.3 (38.8-77.6) | 82.3 (77.0-86.9) |
| CAR^b)^ and inability to ambulate | 0.761 (0.706-0.810) | >0.09 | 74.1 (53.7-88.9) | 67.5 (61.3-73.3) |

a) CRP, C-reactive protein

b) CAR, C-reactive protein to albumin ratio

c) AUC, Area under the curve

d) CI, Confidence interval

Supplementary table 2. Comparison of area under the curve between predictive models related to 28-day mortality

|  | AUC^c)^ (95% CI^d)^) | *P*-value between models | | | |
| --- | --- | --- | --- | --- | --- |
|  |  | CAR^b)^ and inability to ambulate | CRP^a)^ and inability to ambulate | Albumin, CAR^b)^ and inability to ambulate | CRP^a)^, albumin and inability to ambulate |
| CRP^a)^, albumin and inability to ambulate | 0.762 (0.707-0.811) | 0.909 | 0.601 | 0.851 |  |
| Albumin, CAR^b)^ and inability to ambulate | 0.763 (0.708-0.812) | 0.794 | 0.535 |  |  |
| CRP^a)^ and inability to ambulate | 0.750 (0.695-0.800) | 0.486 |  |  |  |
| CAR^b)^ and inability to ambulate | 0.761 (0.706-0.810) |  |  |  |  |

a) CRP, C-reactive protein

b) CAR, C-reactive protein to albumin ratio

c) AUC, Area under the curve

d) CI, Confidence interval
